# Pregnancy and laparoscopic cholecystectomy

**DOI:** 10.4103/0972-9941.45210

**Published:** 2008

**Authors:** Anjum Malik, Iqbal Saleem Mir

**Affiliations:** Lala-Ded Hospital of Obstetrics and Gynaecology, Srinagar, Kashmir, India; 1 Government Medical College, Srinagar, Kashmir, India

Dear Editor,

We read with interest the original article on the feasibility of laparoscopic cholecystectomy(LC) during pregnancy published in the Jan-Mar 2008.[1] There are some points which we would like to elucidate regarding this article.

1. The authors documented six patients who underwent LC during pregnancy. In the abstract the authors comment that two patients were in the first trimester of pregnancy and one of them underwent an elective termination of pregnancy after undergoing LC. However, the case no. two (12 weeks gestation) and case no. six (six weeks gestation) reveal that in both cases healthy babies were delivered at term. In case no two the baby was delivered by caesarean section while as case no. six is silent about the mode of delivery. In the Table 1 and the results section it is again documented that all pregnancies were carried to term. There seems to be a total lack of coherence between the statement made in the abstract and the case histories. What happened to the termination of pregnancy? Did the pregnancy carry to term even after termination? In the abstract there is no mention about the caesarean section yet the authors document the same in case no two. The authors need to clarify these points.
2. In the abstract the authors mention that there was no patient who underwent LC in the third trimester. However in the material and methods they document the gestational age as 17,12,30,13,15 and six weeks. As per standard text books of obstetrics gestation at 30 weeks is considered to be third trimester of pregnancy. If this is a typographical mistake it should be corrected because the essence of the manuscript is lost by this simple mistake.
3. In the case history of patient no. six the authors document that the surgeon was not aware that the patient was pregnant. This amounts to professional negligence as proper history has not been elucidated. Was the patient subjected to any radiological procedure like X-rays or given any drugs which could have deleterious effects on the developing foetus as no shields must have been used. Why was the pregnancy carried to term? How was the surgeon sure that there would be no physical or developmental defects?
4. For how long did the authors follow these children to note any developmental defects? This is a very important aspect which has been left out by the authors.
5. The paragraphs regarding the general contraindications and advantages of laparoscopy are proven facts. These paragraphs have no relevance to the topic.
6. In the paragraph regarding radiological techniques the authors need to elucidate about the requirement of intra operative cholangiography for diagnosis. Are the authors talking about presence of choledocholithisis or biliary tract injuries?

## References

[1] Amoli HA, Tavakoli H, Notash AY, Sajadfar M, Kashayar P (2008). Laparoscopic cholecystectomy during pregnancy: A case series. J Min Access Surg.

